# Berardinelli-Seip syndrome in a Chinese boy with Seipin gene mutation: a case study and literature review of genotype-phenotype

**DOI:** 10.1186/1687-9856-2013-S1-P47

**Published:** 2013-10-03

**Authors:** Shan Huang, Cai Zhang, Yan Liang, Qin Ning, Xiao-Ping Luo

**Affiliations:** 1 Department of Pediatrics, Tongji Hospital. Tongji Medical College of Huazhong University of Science and Technology, Wuhan, China; 2 Department of Infectious Diseases, Tongji Hospital. Tongji Medical College of Huazhong University of Science and Technology, Wuhan, China

## Objective

Congenital generalized lipodystrophy (CGL), or Berardinelli-Seip syndrome, is a rare and heterogeneous disease of autosomal recessive inheritance characterized by the generalized absence of adipose tissue at birth and severe adverse metabolic consequences. The identified causative genes for CGL include 1-acylglycerol-3-phosphate O-acyltransferase 2 (*AGPAT2*), Berardinelli-Seip congenital lipodystrophy 2 (*BSCL2* or Seipin), Caveolin-1 (*CAV1*) and polymerase I and transcript release factor (*PTRF*). Although more than 60 cases of CGL with different gene mutations have been found in Asian patients, only 7 patients were Chinese. Data are also limited regarding genotype-phenotype analysis in Asian CGL patients. Therefore, we aimed to analyze variations of two identified major causative genes, Seipin and *AGPAT2*, involved in CGL etiology in a mainland Chinese affected family and explore the genotype-phenotype of Berardinelli-Seip syndrome in Asian populations.

## Methods

We report a detailed clinical and genetic analysis of a Chinese boy with CGL who was followed from infancy through preschool. Sequences of the entire coding region of Seipin and *AGPAT2* were examined. Phenotypes in various Asian subtypes were compared and the related literature about Berardinelli-Seip syndrome was reviewed.

## Results

We identified a homozygous frameshift mutation (c.974-975insG) in the Seipin gene in the CGL-affected boy. His parents were heterozygous for the same mutation. No variation was found in the AGPAT2 gene.

## Conclusion

In Asian populations, Seipin and PTRF are the main genes identified to date as being responsible for CGL and Seipin is a major causative gene. Genetic heterogeneity is accompanied by phenotypic heterogeneity.

